# *fRagmentomics*: an R package for integrating cell-free DNA fragment features with mutational status to support liquid biopsy interpretation

**DOI:** 10.1093/bioinformatics/btag152

**Published:** 2026-03-26

**Authors:** Killian Maudet, Juliette Samaniego, Yoann Pradat, Elsa Bernard

**Affiliations:** Université Paris-Saclay, Gustave Roussy, Inserm UMR 1361 Cancer Data Science, IHU PRISM National PRecISion Medicine Center in Oncology, Villejuif, F-94805, France; Université Paris-Saclay, Gustave Roussy, Inserm UMR 1361 Cancer Data Science, IHU PRISM National PRecISion Medicine Center in Oncology, Villejuif, F-94805, France; Université Paris-Saclay, Gustave Roussy, Inserm UMR 1361 Cancer Data Science, IHU PRISM National PRecISion Medicine Center in Oncology, Villejuif, F-94805, France; Université Paris-Saclay, Gustave Roussy, Inserm UMR 1361 Cancer Data Science, IHU PRISM National PRecISion Medicine Center in Oncology, Villejuif, F-94805, France

## Abstract

**Summary:**

Liquid biopsy offers a non-invasive approach to study tumor-derived genetic material circulating in plasma. Beyond genetic alterations, the fragmentomic features of cell-free DNA—such as fragment size, genomic position, and end-motifs—provide valuable insights into the biological and clinical context of DNA release. *fRagmentomics* is a user-friendly R package designed to characterize cfDNA fragments overlapping one or multiple small mutations of any type, starting from an aligned sequencing file (BAM). It supports multiple mutation input formats, accommodates one-based and zero-based genomic conventions, resolves mutation representation ambiguities, and accepts any reference file in FASTA format. For each fragment overlapping a mutation of interest, *fRagmentomics* outputs fragment-level features including its fragment size, end-motifs, and mutational status, along with additional fragment-level or read-level information. The package implements an indel-aware and optionally soft-clip-preserving fragment size computation that improves accuracy over conventional size estimates based solely on aligned positions.

**Availability and implementation:**

*fRagmentomics* is licensed under GNU General Public License v3.0 and available at https://github.com/ElsaB-Lab/fRagmentomics, https://anaconda.org/elsab-lab/r-fragmentomics and https://bioconductor.org/packages/fRagmentomics, with documentation and a tutorial.

**Contact:**

yoann.pradat@gustaveroussy.fr, elsa.bernard@gustaveroussy.fr

**Supplementary information:**

Supplementary data are available at *Bioinformatics* online.

## 1 Introduction

Cell-free DNA (cfDNA) refers to fragmented DNA molecules circulating in bodily fluids such as blood, urine, or cerebrospinal fluid (Thierry *et al.* 2016). Sequencing of plasma cfDNA has transformed cancer care by enabling the detection of circulating tumor DNA (ctDNA) for diagnosis, treatment selection, and disease monitoring (Alix-Panabières and Pantel 2021, Bayle *et al.* 2022, Pascual *et al.* 2022, Ma *et al.* 2024, Bartolomucci *et al.* 2025). Most of the cfDNA originates from hematopoietic cells (Mattox *et al.* 2023), complicating ctDNA interpretation in clinical practice. Comprehensive characterization of cfDNA fragmentation features in relation to mutational data is therefore essential for improved liquid biopsy analysis and interpretation.

Fragmentomics—the study of cfDNA fragmentation patterns—examines features such as fragment size, end-motifs, and preferred genomic ends (Tsui *et al.* 2025). Multiple studies have demonstrated that ctDNA fragments have a shorter size distribution (Snyder *et al*. 2016, Mouliere *et al.* 2018, Cristiano *et al.* 2019, Sanchez *et al.* 2021) and distinct end-motif composition (Chan *et al.* 2016, Sun *et al.* 2019, Jiang *et al.* 2020, Shen *et al.* 2024) compared to cfDNA from hematopoietic cells or other origins. CfDNA size distributions can be quantified using non-sequencing approaches, such as electrophoresis- or microfluidics-based methods (Andriamanampisoa *et al.* 2018), which provide global fragment size profiles but do not allow fragment-level genotyping. In contrast, high-depth cfDNA sequencing enables joint analysis of fragmentation patterns and somatic mutations. Recent tools, such as cfDNAPro (Wang *et al.* 2025), combine fragment size analysis with genotyping of selected variants but are limited to specific mutation types and rely on TLEN-based fragment size estimates, which do not account for indels or soft-clipped bases. Consequently, no existing tools provide an integrated solution to both extract fragmentomic features and determine fragment-level mutation status across all classes of small variants, including single-nucleotide variants (SNVs), multi-nucleotide variants (MNVs), and insertions/deletions (indels). This gap partly reflects the technical challenge of indel-aware fragment genotyping: indels can have multiple valid representations (Tan *et al.* 2015), and short reads often fail to fully span indel contexts (Treangen and Salzberg 2011, Narzisi and Schatz 2015). These limitations are particularly relevant given that indels include clinically actionable biomarkers, such as *EGFR* exon 19 deletions predicting response to EGFR inhibitors in non-small-cell lung cancer (Lu *et al.* 2024), and that inaccurate fragment size estimation may confound downstream fragmentomic analyses.

*fRagmentomics* is a standardized and user-friendly R package that extracts per-fragment features (size, end-motifs, genomic coordinates) and assigns mutation status for any small variant type. Its indel-aware fragment size calculation ensures greater accuracy than conventional reference-span metrics. By enabling mutation-aware exploration of cfDNA fragmentation patterns, *fRagmentomics* may facilitate the biological and clinical interpretation of liquid biopsy sequencing data.

## 2 Methods

**Figure 1:**
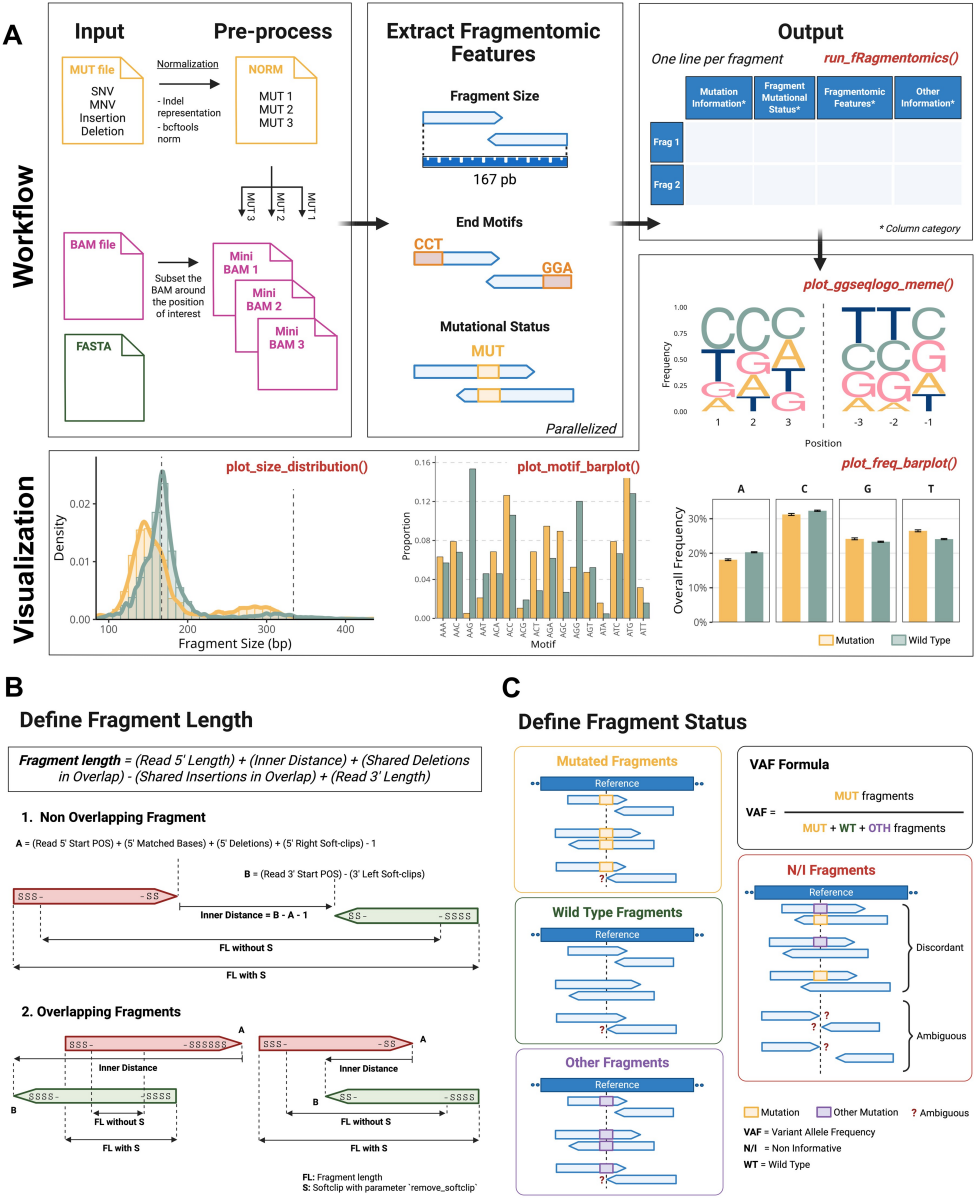
**Overview of the *fRagmentomics* workflow and core computations. (A) Workflow summary**. *fRagmentomics* takes a BAM file and a list of small mutations as input and outputs a table with one row per fragment. Each row contains mutation information, fragmentomic features, and optional read-level details. Built-in visualization functions enable exploration of fragment size distributions, end-motifs, and mutation patterns. The plots shown were generated using the example dataset included in the package. **(B) Fragment size computation**. Fragment length is computed using an indel-aware formula that accounts for insertions, deletions, and soft-clipped bases, providing more accurate size estimates than TLEN-based methods. **(C) Fragment genotyping**. Each fragment is classified as *mutated* (MUT), *wild-type* (WT), *other* (OTH), or *non-informative* (N/I; ambiguous or discordant) based on read-level genotypes derived from sequence comparison and CIGAR information. The fragment-level variant allele frequency (VAF) is calculated as the ratio of mutated fragments to the total number of informative fragments.

*fRagmentomics* is implemented in R and organized around a single high-level function, *run_fRagmentomics()*, which produces a per-fragment table with one row per fragment and one column per feature. For each user-provided mutation, the function performs three main steps (Fig. 1A): (i) input preparation and fragment selection, (ii) fragmentomic feature extraction, and (iii) genotyping of reads and fragments. The latter two steps are parallelized for efficiency. The resulting table can be used directly for downstream analyses or visualization with built-in plotting functions. Various options allow the user to fine-tune the behavior of each step. All parameters and default values are summarized in Supplementary Table S1, available as supplementary data at *Bioinformatics* online.

### 2.1 Mutation and BAM file preparation

*fRagmentomics* requires three mandatory inputs: (i) a BAM file with paired-end reads, (ii) a list of mutations provided as a VCF/TSV file or as “chr:pos:ref:alt” strings, and (iii) the reference genome used for alignment. To ensure consistency across different mutation formats and conventions (Supplementary Methods S1, available as supplementary data at *Bioinformatics* online), the package automatically normalizes each mutation through three steps: handling missing allele representations, harmonization to VCF standards, and optionally calling *bcftools norm* to resolve representation ambiguities (Tan *et al.* 2015, Danecek *et al.* 2021).

The BAM file is then subsetted to fragments overlapping the genomic region of each mutation, within a user-defined window set by default to ±600 bp. Fragments not satisfying user-configurable BAM flags or those failing quality checks are excluded (Supplementary Methods S2, available as supplementary data at *Bioinformatics* online). This restriction balances runtime efficiency and fragment recovery, with default parameters sufficient to capture the full distribution of human cfDNA fragment lengths (Snyder *et al*. 2016).

### 2.2 Extraction of fragmentomic features

For each mutation of interest, *fRagmentomics* computes fragment-level characteristics in a loop parallelized across individual fragments. The complete list of extracted features is provided in Supplementary Table S2, available as supplementary data at *Bioinformatics* online, and includes several key components.

The fragment size is computed in an indel-aware and soft-clip-preserving manner (Supplementary Methods S3, Supplementary Figure S1, available as supplementary data at *Bioinformatics* online) and represents the true physical length of the cfDNA fragment. Explicit handling of indels prevents under- or over-estimation of fragment size in the presence of such variants. Fragments shorter than the read length are accurately measured through the inner distance calculation (Fig. 1B, Supplementary Figure S1, available as supplementary data at *Bioinformatics* online). Users may optionally discard soft-clipped bases according to their assumptions about the biological or technical origin of soft-clipping (Supplementary Figure S2, available as supplementary data at *Bioinformatics* online). End-motifs (5′ and 3′ sequences) are extracted from the reference, with a default length of 5 bases. The fragment‘s first aligned and last aligned positions, the inner distance between reads, and the counts of soft-clipped bases at the 5′ and 3′ ends are also reported. Optional read-level attributes from the BAM such as CIGAR, FLAGs, and base qualities can be included for diagnostic purposes or downstream analyses. Numerous user-adjustable parameters allow fine-tuning of fragment extraction, soft-clip handling, motif length, and output options (Supplementary Table S1, available as supplementary data at *Bioinformatics* online).

To demonstrate the importance of indel-aware computation, we compared *fRagmentomics* with cfDNAPro (Wang *et al.* 2025), for which fragment sizes are equal to the BAM TLEN field. We first analyzed 10 SNVs/MNVs in targeted cfDNA sequencing samples. A total of 296,025 out of 299,245 fragments (98.9%) showed identical sizes between the two tools. Those with discordant sizes had a mean absolute difference of 1.5 bp (range [–36; +35]), all attributable to the presence of indels within the CIGAR string. We then evaluated 10 small indels (three 1-bp deletions, one 5-bp deletion, one 4-bp deletion, four 1-bp insertions, and one 3-bp insertion). Of 233,998 common fragments, 230,317 (98.4%) had identical sizes, with discordant cases showing a mean absolute difference of 1.7 bp (range [–5; +5]). When focusing on mutated fragments, 1,999 of 2,001 (99.9%) were discordant, with a mean absolute difference of 1.5 bp (range [–36; +35]). Finally, we analyzed a lung cancer cfDNA sample (included in the package test dataset) harboring an *EGFR* exon 19 in-frame deletion (15 bp) (Supplementary Figure S3, available as supplementary data at *Bioinformatics* online). Out of 3,017 common fragments, 2,217 (73.5%) showed identical sizes between tools. However, all mutated fragments (26.2%) were discordant, with an absolute size difference of 15 bp (range [14; 15]), corresponding to the deletion length. These comparisons highlight that while TLEN-based fragment size calculation is generally sufficient for fragments harboring SNVs/MNVs, it remains inadequate for those carrying indels, emphasizing the need for indel-aware fragment size computation.

### 2.3 Read and fragment genotyping

Mutation status is assigned at both the read and fragment levels. For each read overlapping a mutation, the read sequence over a local window (Supplementary Figure S4, available as supplementary data at *Bioinformatics* online) is compared to the reference allele sequence (extracted from the FASTA) and to the mutated allele sequence (constructed by applying the mutation to the reference sequence). The definition of the window varies according to the mutation type (Supplementary Methods S4.1, available as supplementary data at *Bioinformatics* online). For SNVs/MNVs, a first window covers the position of the substituted base and a second window, extended by one flanking base on each side, flags potentially larger events. For indels, the window includes one base preceding the indel, the inserted bases in case of an insertion, one base following the indel, and additional bases if necessary to resolve ambiguities in repetitive contexts. Windows are truncated when reads do not fully cover the defined region. Example window calculations are provided in Supplementary Figure S4, available as supplementary data at *Bioinformatics* online.

The sequence comparison yields a *wild-type*, *mutated*, *other*, or *ambiguous* status (Supplementary Methods S4.2, available as supplementary data at *Bioinformatics* online). This status is directly used to determine the read-level mutation assignment for SNVs and MNVs (Supplementary Methods S4.3, available as supplementary data at *Bioinformatics* online), while indel genotyping integrates both the CIGAR and sequence comparison, giving precedence to CIGAR information. When no indel is reported at the position of interest, only the sequence comparison is used. Flags are added to the read status to indicate potential ambiguities, inconsistencies between the CIGAR and the sequence comparison, or the presence of another variant (Supplementary Methods S4.4, available as supplementary data at *Bioinformatics* online).

Read-level statuses are then aggregated into a fragment-level status (Fig. 1C). Concordant reads define the fragment status directly. Discordant reads are resolved using flags when possible (Supplementary Figure S3.D, available as supplementary data at *Bioinformatics* online). Fragments that cannot be resolved are labeled *discrepant*. For simplicity, *discrepant* and *ambiguous* fragments are combined into a non-informative category (Supplementary Methods S5, available as supplementary data at *Bioinformatics* online). Non-informative fragments are excluded from fragment-level variant allele frequency calculations, which use only informative fragments. The dual-level genotyping ensures robust mutation assignment while retaining detailed information on uncertainty, misalignments, and potential alternate events.

## 3 Output and visualization

*fRagmentomics* produces a comprehensive fragment-level table where each row corresponds to a fragment overlapping one of the user-specified mutations. Columns include fragment-level features (e.g., size, start and end-motifs, genomic coordinates, soft-clip counts, genotyping status) and optional read-level attributes such as FLAGs, mapping qualities, and CIGAR strings (Supplementary Table S2, available as supplementary data at *Bioinformatics* online).

A set of built-in visualization functions allows rapid exploration of fragmentomic patterns directly from the generated table (Fig. 1A). Each function provides options to choose how fragments are grouped and which groups are displayed. The *plot_size_distribution()* function generates fragment-size profiles as either density curves or histograms. The *plot_motif_barplot()* function supports three representation modes of the motif sequences to allow for comparative or differential views. The *plot_freq_barplot()* function compares nucleotide composition at fragment ends across groups, regardless of motif position, while *plot_ggseqlogo_meme()* produces sequence logos showing nucleotide proportions at each motif position.

All plotting functions return *ggplot2* objects for easy customization and integration into analysis workflows. Example scripts reproducing Fig. 1A are provided in the vignette and in the package’s GitHub repository.

The algorithm operates with a complexity of O(n x L) per mutation, with n the number of fragments overlapping the mutation of interest and L the read length. Benchmarking at various read depths demonstrated efficient performance (Supplementary Figure S5, available as supplementary data at *Bioinformatics* online). The test dataset accompanying the package, containing about 3,000 fragments, was processed in 36 seconds with a peak memory usage below 1 GB on a single-core Intel Xeon E5-2670 v3 processor. To further optimize runtime, the implementation supports parallel processing over fragments, which can be activated by providing multiple CPUs.

## 4 Conclusion

*fRagmentomics* is a user-friendly R package that integrates per-fragment feature extraction and genotyping across all classes of small mutations (SNVs, MNVs, and indels). It explicitly handles the complexity of indel detection and provides flags to indicate ambiguous or potentially erroneous genotypes. The package implements an indel-aware, soft-clip-preserving fragment-size computation, improving accuracy compared with conventional methods based on aligned extremal positions.

A single high-level function, *run_fRagmentomics()*, executes the full workflow and returns a fragment-level table suitable for downstream analyses and visualization. Users can fine-tune fragmentomic extraction, soft-clip handling, and visualization options, and the tool supports both normalized and unnormalized variant representations.

*fRagmentomics* is freely available on GitHub at https://github.com/ElsaB-Lab/fRagmentomics with a detailed tutorial. It has also been deposited on Conda and Bioconductor.

## Supplementary Material

btag152_Supplementary_Data

## Data Availability

The *fRagmentomics* R package, along with the test dataset used to generate the vignette, is publicly available on GitHub (https://github.com/ElsaB-Lab/fRagmentomics), Bioconductor (https://bioconductor.org/packages/fRagmentomics), and archived on Zenodo (DOI: 10.5281/zenodo.18679583). Test dataset files used for demonstration and figures are provided under the directory *inst/extdata*. The scripts used to create and anonymize the test dataset are available in *inst/scripts.* All code used for figure generation, example workflows, and supplementary analyses is included in the Supplementary Methods, package vignette, and documentation. *fRagmentomics* is distributed under the GNU General Public License v3.0.

## References

[btag152-B1] Alix-Panabières C , Pantel K. Liquid biopsy: From discovery to clinical application. Cancer Discov 2021;11:858–73. 10.1158/2159-8290.CD-20-1311 33811121

[btag152-B2] Andriamanampisoa C-L , Bancaud A, Boutonnet-Rodat A et al BIABooster: Online DNA concentration and size profiling with a limit of detection of 10 fg/μl and application to high-sensitivity characterization of circulating cell-free DNA. Anal Chem 2018;90:3766–74. 10.1021/acs.analchem.7b04034 29498256

[btag152-B3] Bartolomucci A , Nobrega M, Ferrier T et al Circulating tumor DNA TO monitor treatment response in solid tumors and advance precision oncology. NPJ Precis Oncol 2025;9:84. 10.1038/s41698-025-00876-y 40122951 PMC11930993

[btag152-B4] Bayle A , Peyraud F, Belcaid L et al Liquid versus tissue biopsy for detecting actionable alterations according to the ESMO scale for clinical actionability of molecular targets in patients with advanced cancer: A study from the french national center for precision medicine (PRISM). Ann Oncol 2022;33:1328–31. 10.1016/j.annonc.2022.08.089 36122799

[btag152-B5] Chan KCA , Jiang P, Sun K et al Second generation noninvasive fetal genome analysis reveals de novo mutations, single-base parental inheritance, and preferred DNA ends. Proc Natl Acad Sci U S A 2016;113:E8159–68. 10.1073/pnas.1615800113 27799561 PMC5167168

[btag152-B6] Cristiano S , Leal A, Phallen J et al Genome-wide cell-free DNA fragmentation in patients with cancer. Nature 2019;570:385–9. 10.1038/s41586-019-1272-6 31142840 PMC6774252

[btag152-B7] Danecek P , Bonfield JK, Liddle J et al Twelve years of samtools and bcftools. Gigascience 2021;10:giab008. 10.1093/gigascience/giab008 33590861 PMC7931819

[btag152-B8] Jiang P , Sun K, Peng W et al Plasma DNA end-motif profiling as a fragmentomic marker in cancer, pregnancy, and transplantation. Cancer Discov 2020;10:664–73. 10.1158/2159-8290.CD-19-0622 32111602

[btag152-B9] Lu S , Kato T, Dong X et al Osimertinib after chemoradiotherapy in stage III EGFR-mutated NSCLC. N Engl J Med 2024;391:585–97. 10.1056/NEJMoa2402614 38828946

[btag152-B10] Ma L , Guo H, Zhao Y et al Liquid biopsy in cancer current: status, challenges and future prospects. Signal Transduct Target Ther 2024;9:336. 10.1038/s41392-024-02021-w 39617822 PMC11609310

[btag152-B11] Snyder MW , Kircher M, Hill AJ et al Cell-free DNA comprises an in vivo nucleosome footprint that informs its tissues-of-origin. Cell 2016;164:57–68. 10.1016/j.cell.2015.11.050 26771485 PMC4715266

[btag152-B12] Mattox AK , Douville C, Wang Y et al The origin of highly elevated cell-free DNA in healthy individuals and patients with pancreatic, colorectal, lung, or ovarian cancer. Cancer Discov 2023;13:2166–79. 10.1158/2159-8290.CD-21-1252 37565753 PMC10592331

[btag152-B13] Mouliere F , Chandrananda D, Piskorz AM et al Enhanced detection of circulating tumor DNA BY fragment size analysis. Sci Transl Med 2018;10:eaat4921. 10.1126/scitranslmed.aat4921 30404863 PMC6483061

[btag152-B14] Narzisi G , Schatz MC. The challenge of small-scale repeats for indel discovery. Front Bioeng Biotechnol 2015;3:8. 10.3389/fbioe.2015.00008 25674564 PMC4306302

[btag152-B15] Pascual J , Attard G, Bidard F-C et al ESMO recommendations on the use of circulating tumour DNA assays for patients with cancer: A report from the ESMO precision medicine working group. Ann Oncol 2022;33:750–68. 10.1016/j.annonc.2022.05.520 35809752

[btag152-B16] Sanchez C , Roch B, Mazard T et al Circulating nuclear DNA structural features, origins, and complete size profile revealed by fragmentomics. JCI Insight 2021;6:e144561. 10.1172/jci.insight.144561 33571170 PMC8119211

[btag152-B17] Shen H , Yang M, Liu J et al Development of a deep learning model for cancer diagnosis by inspecting cell-free DNA end-motifs. NPJ Precis Oncol 2024;8:160. 39068267 10.1038/s41698-024-00635-5 PMC11283569

[btag152-B18] Sun K , Jiang P, Cheng SH et al Orientation-aware plasma cell-free DNA fragmentation analysis in open chromatin regions informs tissue of origin. Genome Res 2019;29:418–27. 10.1101/gr.242719.118 30808726 PMC6396422

[btag152-B19] Tan A , Abecasis GR, Kang HM. Unified representation of genetic variants. Bioinformatics 2015;31:2202–4. 10.1093/bioinformatics/btv112 25701572 PMC4481842

[btag152-B20] Thierry AR , El Messaoudi S, Gahan PB et al Origins, structures, and functions of circulating DNA in oncology. Cancer Metastasis Rev 2016;35:347–76. 10.1007/s10555-016-9629-x 27392603 PMC5035665

[btag152-B21] Treangen TJ , Salzberg SL. Repetitive DNA AND next-generation sequencing: computational challenges and solutions. Nat Rev Genet 2011;13:36–46. 10.1038/nrg3117 22124482 PMC3324860

[btag152-B22] Tsui WHA , Jiang P, Lo YMD. Cell-free DNA fragmentomics in cancer. Cancer Cell 2025;43:1792–814. 10.1016/j.ccell.2025.09.006 41043439

[btag152-B23] Wang H , Mennea PD, Chan YKE et al A standardized framework for robust fragmentomic feature extraction from cell-free DNA sequencing data. Genome Biol 2025;26:141. 10.1186/s13059-025-03607-5 40410787 PMC12100915

