# Supplementary material for: fRagmentomics: an R package for integrating cell-free DNA fragment features with mutational status to support liquid biopsy interpretation
Source: Bioinformatics. 2026 Mar 26;42(4):btag152. doi: 10.1093/bioinformatics/btag152 (PMC13070676; doi:10.1093/bioinformatics/btag152)
Supplement: btag152_Supplementary_Data [file btag152_supplementary_data.zip › 31-Mar-2026_020819_Supplementary_fRagmentomics_final.pdf]

Supplementary Material

fRagmentomics: an R package for integrating cell-free DNA fragment features with mutational status to support liquid biopsy interpretation.

Killian Maudet[1], Juliette Samaniego[1], Yoann Pradat[#,1] and Elsa Bernard[#,1]

1. Université Paris-Saclay, Gustave Roussy, Inserm UMR 1361 Cancer Data Science, IHU PRISM National PRecISion Medicine Center in Oncology, Villejuif F-94805, France

Supplementary Tables
- Supplementary Table S1. List of complete default parameters.
- Supplementary Table S2. Output table.

Supplementary Figures
- Supplementary Figure S1. Illustration of fragment size formula with examples.
- Supplementary Figure S2. Impact of the Remove_softclip parameter on fragment size calculation.
- Supplementary Figure S3. Application example using an EGFR exon 19 deletion case.
- Supplementary Figure S4. Example of comparison windows.
- Supplementary Figure S5. fRagmentomics time and memory consumption.

Supplementary Methods
- S1. Mutation input normalization
- S2. Read selection and windowing
- S3. Fragment size computation
- S4. Read genotyping
  - S4.1 Window definition
  - S4.2 Sequence comparison
  - S4.3 SNVs/MNVs genotyping
  - S4.4 Indels genotyping
- S5. Fragment genotyping
- S6. Commands to reproduce outputs and plots in Fig.1

Supplementary References

Supplementary Tables

Supplementary Table S1. List of complete default parameters.

Parameter	Description	Default
**mut**	Path to a .vcf or .tsv file or string representation chr:pos:ref:alt of a mutation.	NEEDED
**bam**	Path to a BAM file.	NEEDED
**fasta**	Path to the FASTA file for the reference sequence used for generating the BAM file.	NEEDED
**sample_id**	Sample identifier.	NA
**neg_offset_mate_search**	Integer. Used in read_bam. Represents the number of nucleotides to extend upstream (negative direction) from the position of interest when querying the BAM file with Rsamtools. This extension ensures that paired reads are retrieved, even if only one mate overlaps the queried position.	-600
**pos_offset_mate_search**	Integer. Used in read_bam. Same as neg_offset_mate_search.	+600
**one_based**	Boolean. TRUE if fasta is one based. False if 0 based.	TRUE
**flag_bam_list**	A named list of logicals for filtering reads based on their SAM flag.<br>NA = Filter is ignored,<br>TRUE = The read MUST have this flag,<br>FALSE = The read MUST NOT have this flag.	isPaired = TRUE,<br>isProperPair = NA<br>isUnmappedQuery = FALSE<br>hasUnmappedMate = FALSE<br>isMinusStrand = NA<br>isMateMinusStrand = NA<br>isFirstMateRead = NA<br>isSecondMateRead = NA<br>isSecondaryAlignment = FALSE<br>isSupplementaryAlignment = FALSE,<br>isNotPassingQualityControls = NA,<br>isDuplicate = NA
**report_bam_info**	Boolean. Whether to include the bam information.	FALSE
**report_softclip**	Boolean. Whether to include the number of soft-clipped bases at	FALSE

	the fragment extremities in the output.	
**report_5p_3p_ bases_fragment**	Integer. Whether to include N fragment extremity bases in the output.	5
**remove_softclip**	Boolean. If set to TRUE, remove all soft-clipped bases.	FALSE
**retain_fail_qc**	Boolean. If set to TRUE, retain fragments that failed the various quality checks in the output.	FALSE
**apply_bcftools_ norm**	Boolean. If set to TRUE, apply bcftools norm on each input variant to normalize it. Require that bcftools command is installed and available in the PATH.	FALSE<br><br>WARNING for indels, highly recommended to set TRUE
**tmp_folder**	Character vector for the temporary folder path.	tempdir()
**output_path**	Character vector for the fragmentomics table output path. If set to TRUE, the function returns NULL.	NA
**verbose**	Boolean. If set to TRUE, print all the warnings and the prints.	FALSE
**n_cores**	Number of cores for parallel computation.	1

Supplementary Table S2. Output table.

Each row of the resulting table corresponds to one fragment and includes the following columns:

**Column**	**Description**
**Mutation Information**	
Sample_Id	User-provided sample identifier.
Chromosome	Chromosome of the mutation **after** normalization.
Position	Start position of the mutation **after** normalization.
Ref	Reference allele **after** normalization.
Alt	Alternate allele **after** normalization.
Input_Mutation	The original mutation information as provided in the input file.
**Fragment & Read Status**	
Fragment_Id	The read name (QNAME) that uniquely identifies the DNA fragment.
Fragment_QC	Quality control status. Is "OK" for valid pairs or contains a failure reason.
Fragment_Status_Simple	Simplified mutation status of the fragment ("MUT", "WT", "OTH", "N/I").
Fragment_Status_Detail	Detailed mutation status, created by concatenating read statuses if they differ.
Read_5p_Status	Mutation status for the 5' read ("MUT", "WT", "OTH", "AMB", "[MUT/WT/OTH] by CIGAR but potentially [MUT/WT/OTH]").
Read_3p_Status	Mutation status for the 3' read ("MUT", "WT", "OTH", "AMB", "[MUT/WT/OTH] by CIGAR but potentially [MUT/WT/OTH]").
BASE_5p	Base(s) from the 5' read covering the variant position.
BASE_3p	Base(s) from the 3' read covering the variant position.
BASQ_5p	Base quality/qualities from the 5' read covering the variant position.
BASQ_3p	Base quality/qualities from the 3' read

	covering the variant position.
**Fragmentomic & Alignment Features**	
Fragment_Size	The size of the DNA fragment.
Position_5p	1-based leftmost mapping position of the 5' read.
Position_3p	1-based rightmost mapping position of the 3' read.
Fragment_Bases_5p (if `report_5p_3p_bases_fragment` > 0)	The first n bases from the 5' end of the fragment.
Fragment_Bases_3p (if `report_5p_3p_bases_fragment` > 0)	The last n bases from the 3' end of the fragment.
**Bam Information** (if `report_bam_info` = TRUE)	
POS_5p	1-based leftmost mapping position of the 5' read.
POS_3p	1-based leftmost mapping position of the 3' read.
FLAG_5p	SAM flag for the 5' read.
FLAG_3p	SAM flag for the 3' read.
MAPQ_5p	Mapping quality for the 5' read.
MAPQ_3p	Mapping quality for the 3' read.
CIGAR_5p	CIGAR string for the 5' read.
CIGAR_3p	CIGAR string for the 3' read.
TLEN	Template length of the fragment, from the BAM file.
**Other Information**	
VAF	Variant Allele Frequency, expressed as a percentage.
Fragment_Basqs_5p (if `report_5p_3p_bases_fragment` > 0)	The first n base qualities from the 5' end of the fragment.
Fragment_Basqs_3p (if `report_5p_3p_bases_fragment` > 0)	The first n base qualities from the 3' end of the fragment.
Nb_Fragment_Bases_Softclip_5p (if `report_softclip` = TRUE)	Number of soft-clipped bases at the 5' end of the fragment.

Nb_Fragment_Bases_Softclip_3p (if `report_softclip` = TRUE)	Number of soft-clipped bases at the 3' end of the fragment.

Supplementary Figures

Supplementary Figure S1. Illustration of fragment size formula with examples.

Formula

Fragment Size = (Read 5' Length) + (Inner Distance) + (Shared Deletions in Overlap) - (Shared Insertions in Overlap) + (Read 3' Length)

1. **Inner Distance** = (Read 3' Inner Boundary) - (Read 5' Inner Boundary) - 1
2. **Read 5' Inner Boundary** = (Read 5' first aligned position) + (5' Matched Bases) - 1 + (5' Deletions) + (5' Right Soft-clips)
3. **Read 3' Inner Boundary** = (Read 3' first aligned position) - (3' Left Soft-clips)

A = Read 5' Inner Boundary: Reference-based position. Consequently, the Inner Distance reflects the genomic span on the reference and **does not account for** physical indels within the fragment's gap.
B = Read 3' Inner Boundary: Same logic as Read 5' Inner Boundary.

Case 1: Fragment size calculation with an insertion outside the overlap

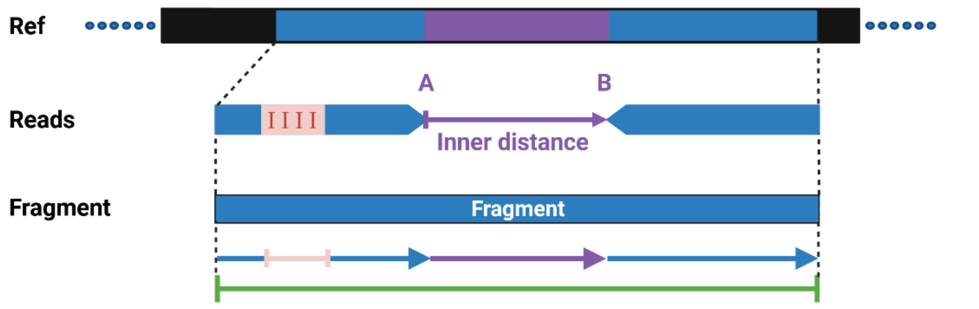

Fragment Size = (**Read 5' Length**) + (**Inner Distance**) + (Shared Deletions in Overlap) - (Shared Insertions in Overlap) + (**Read 3' Length**)

with (Shared Deletions in Overlap) and (Shared Insertions in Overlap) **= 0**
and (Inner Distance) **> 0** because B > A

Case 2: Fragment size calculation with an insertion inside the overlap

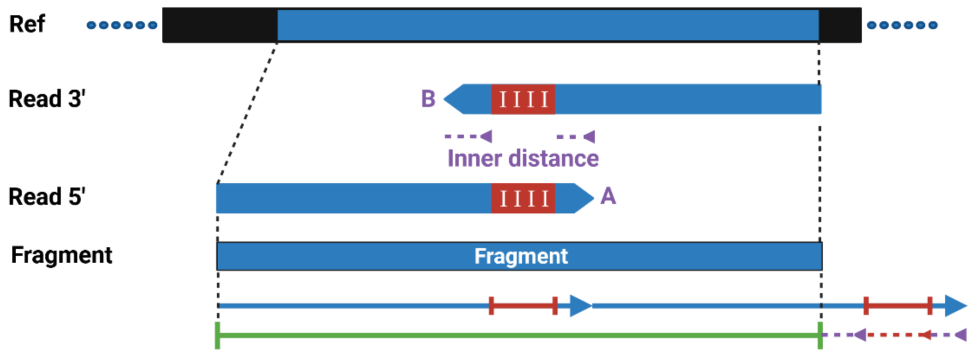

Fragment Size = (**Read 5' Length**) + (**Inner Distance**) + (Shared Deletions in Overlap) - (**Shared Insertions in Overlap**) + (**Read 3' Length**)

with (Shared Deletions in Overlap) **= 0**
and (Inner Distance) < **0** because A > B

Case 3: Fragment size calculation with a deletion inside the overlap

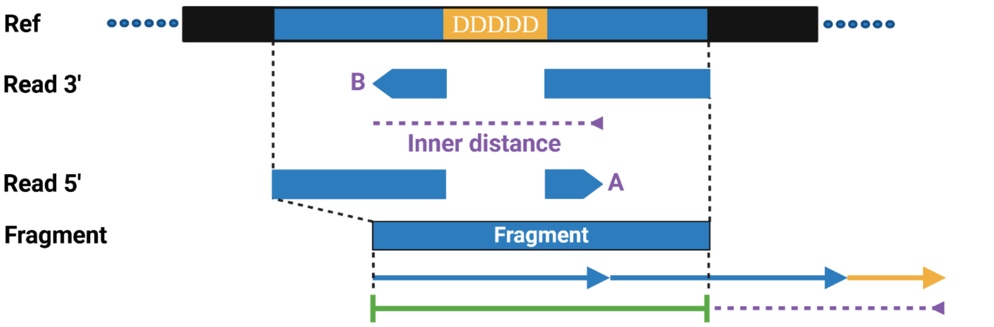

Fragment Size = (Read 5' Length) + (Inner Distance) + (Shared Deletions in Overlap) - (Shared Insertions in Overlap) + (Read 3' Length)

with (Shared Insertions in Overlap) **= 0**
and (Inner Distance) **< 0** because A > B

Case 4: Fragment size calculation with read length > fragment size

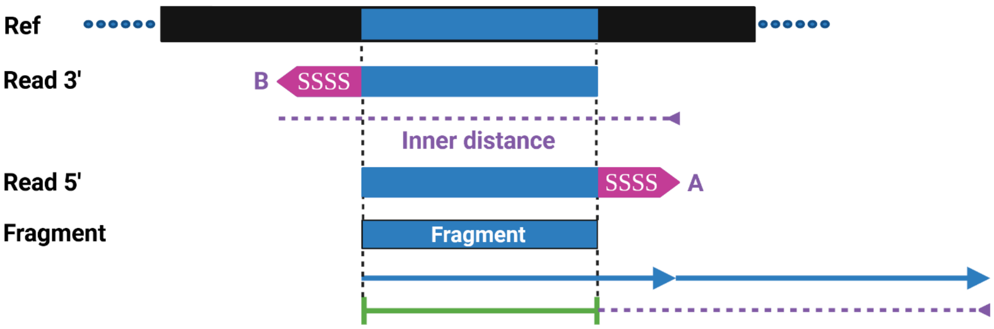

Fragment Size = (Read 5' Length) + (Inner Distance) + (Shared Deletions in Overlap) - (Shared Insertions in Overlap) + (Read 3' Length)

with (Shared Deletions in Overlap) and (Shared Insertions in Overlap) **= 0**
and (Inner Distance) < **0** because A > B

Legend

→ Added to the fragment length formula

◄ (dashed) Subtracted in the fragment length formula

Supplementary Figure S2. Impact of the Remove_softclip parameter on fragment size calculation.

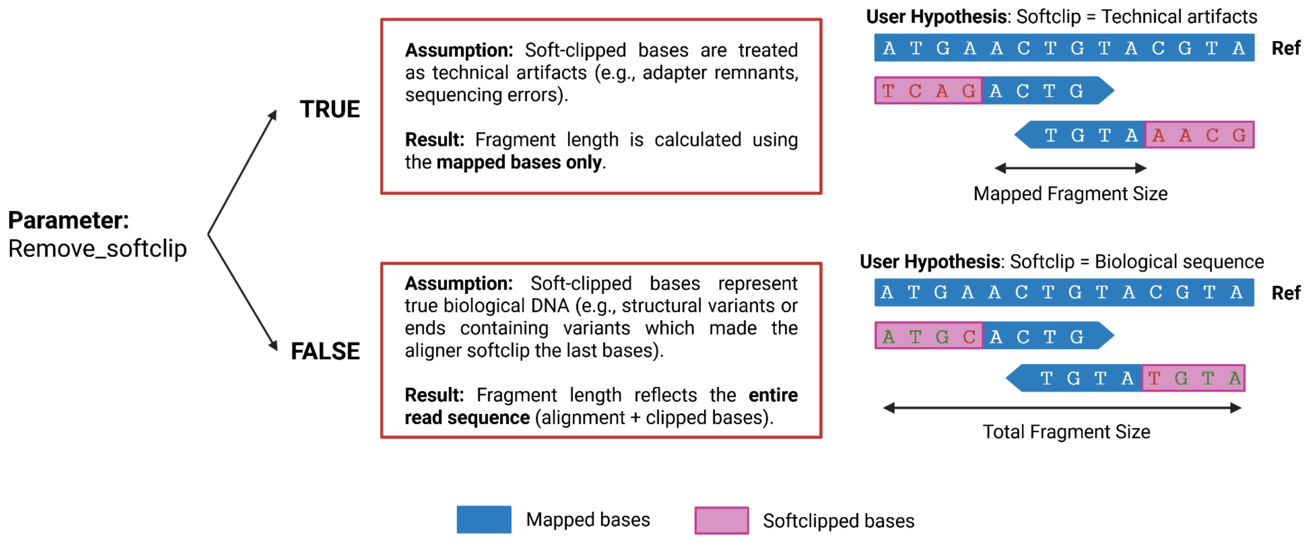

Supplementary Figure S3. Application example using an *EGFR* exon 19 deletion case.

(A) Workflow for generating the test BAM file. To ensure portability, the BAM file (±600 bp) and reference FASTA (±10,000 bp) were subsetted around the mutation site. Genomic coordinates in the BAM were shifted relatively to the start of the FASTA to maintain consistency between the alignments and the FASTA. **(B) Fragment size distribution of mutated (MUT) fragments.** To compare with cfDNAPro, we employed its default settings: flag_bam_list(isPaired=TRUE, isSecondaryAlignment=FALSE, isUnmappedQuery=FALSE, isSupplementaryAlignment=FALSE, isDuplicate=FALSE) and remove_softclip=TRUE. *fRagmentomics* (red) correctly captures the 15-bp physical shift compared to reference-based *cfDNAPro* (blue). **(C) Fragments QC and mutation statuses.** *fRagmentomics* reported 3,024 fragments overlapping the deletion position, of which 7 failed the package internal QC. The 3,017 other fragments were shared with *cfDNAPro*. **(D) Fragment genotyping resolved by sequence comparison.** Example fragments for which the mutation status differs between paired reads but the fragment-level status can be resolved to "MUT". Out of 791 MUT fragments, 762 were MUT on both reads, 28 were MUT on one read and AMB on the other read, and 1 was MUT on one read and "WT by CIGAR but potentially MUT" on the other read. VAF: variant allele frequency; N/I: not informative; OTH: other; AMB: ambiguous.

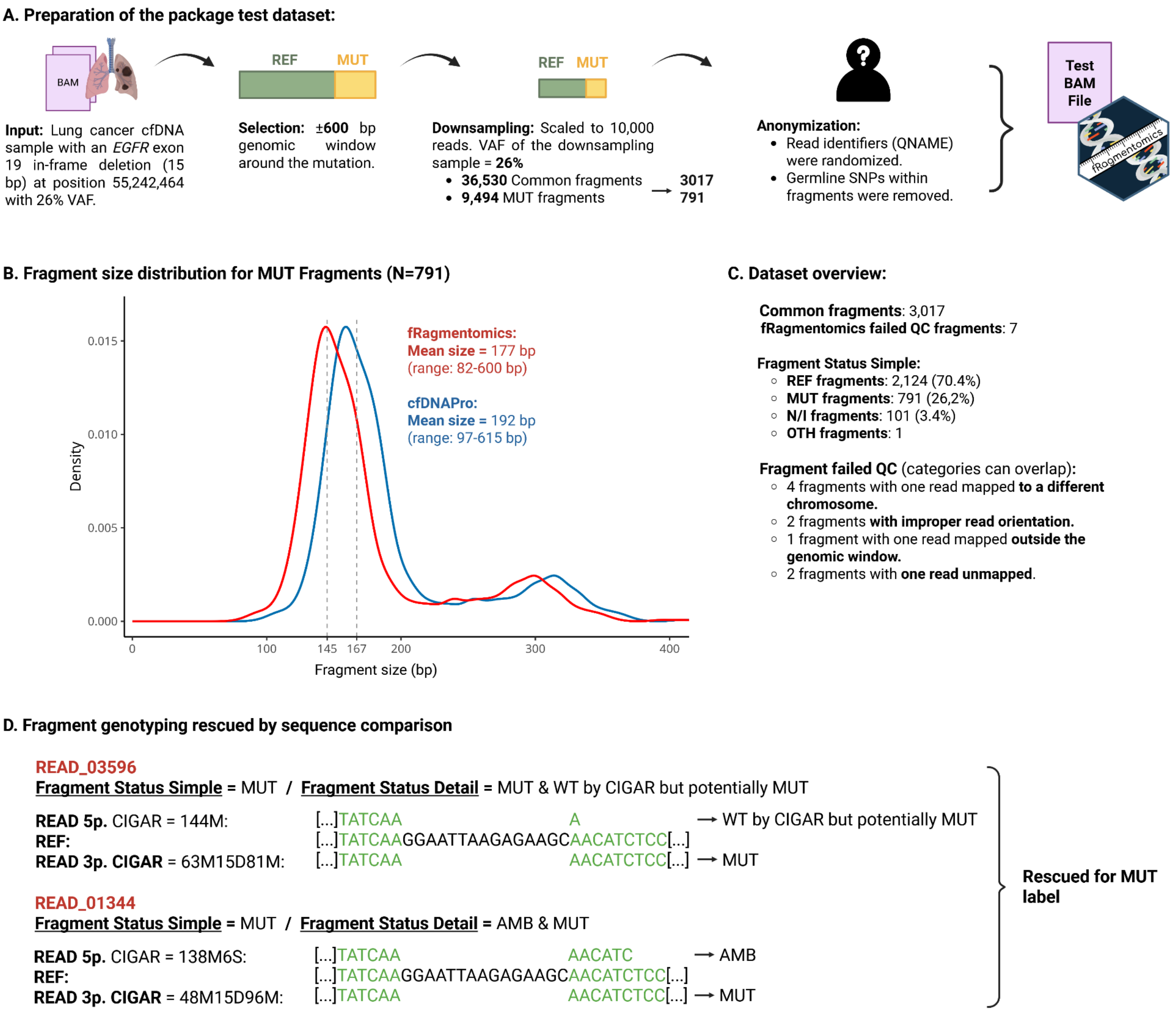

Supplementary Figure S4. Example of comparison windows.

The comparison window defines the portion of the read that is compared to the corresponding region of the reference and mutated sequences. It represents the minimal segment necessary to unambiguously determine whether the read supports the wild-type (WT), mutant (MUT), or another (OTH) allele.

Parameters

n_{before}: The number of upstream anchor bases (default = 1).
R: The number of consecutive occurrences of the indel motif in the reference sequence, including the mutated base itself.
n_{shared}: The number of additional overlapping bases shared with the motif but not forming a complete repeat.
n_{after}: The number of downstream bases (default = 1).

Deletion without repeated sequence

Pos = 2 **Ref** = CA **Alt** = C

Ref sequence = T C A C G T A G. . . **Read sequence** = T C A C G T

1) Define Ref WT and Ref Mut

Reference Sequence WT = C A C G T
Reference Sequence MUT = C C G T

2) Define Windows Comparison

$compare\_len_{WT} = compare\_len_{MUT} = n_{before} + (R-1) \times L_{motif} + n_{shared} + n_{after}$
$compare\_len_{WT} = compare\_len_{MUT} = 1 + (1-1) * 1 + 0 + 1 = \underline{2}$

3) Sequence comparison

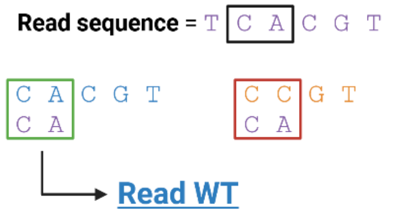

A minimum of **two nucleotides** is required to unambiguously assign the read status = **WT**.

Deletion with repeated sequence

Pos = 2 **Ref** = CAA **Alt** = C

Ref sequence = T C A A A T A G. . . **Read sequence** = T C A T A G

1) Define Ref WT and Ref Mut

Reference Sequence WT = C A A A T
Reference Sequence MUT = C A T

2) Define Windows Comparison

$compare\_len_{WT} = compare\_len_{MUT} = n_{before} + (R-1) \times L_{motif} + n_{shared} + n_{after}$
$compare\_len_{WT} = compare\_len_{MUT} = 1 + (1-1) * 2 + 1 + 1 = \underline{3}$

3) Sequence comparison

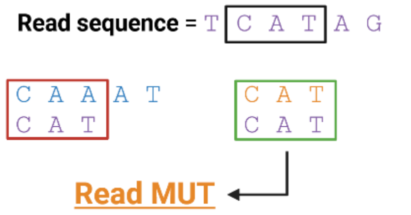

A minimum of **three nucleotides** is required to unambiguously assign the read status = **MUT**.

Insertion with repeated sequence

Pos = 2 **Ref** = C **Alt** = CA

Ref sequence = T C A A G T A G . . .

Read sequence = T C A A A G

1) Define Ref WT and Ref Mut

Reference Sequence WT = C A A G T
Reference Sequence MUT = C A A A G

2) Define Windows Comparison

compare_len$_{WT}$ = $n_{before} + R \times L_{motif} + n_{shared} + n_{after}$
compare_len$_{WT}$ = $1 + 2 * 1 + 0 + 1 =$ <u>**4**</u>

compare_len$_{MUT}$ = $n_{before} + (R+1) \times L_{motif} + n_{shared} + n_{after}$
compare_len$_{MUT}$ = $1 + (2+1) * 1 + 0 + 1 =$ <u>**5**</u>

3) Sequence comparison

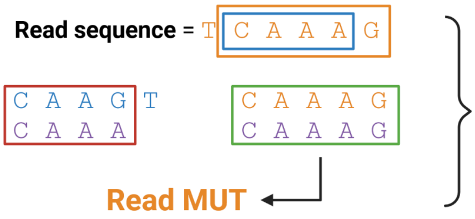

A minimum of **five nucleotides** is required to compare the read with the **reference Sequence MUT** and **four nucleotides** to compare with the **reference Sequence WT**.

Read status = **MUT**

Supplementary Figure S5. *fRagmentomics* time and memory consumption.

Time (left) and memory (right) consumptions as a function of the number of fragments analyzed.

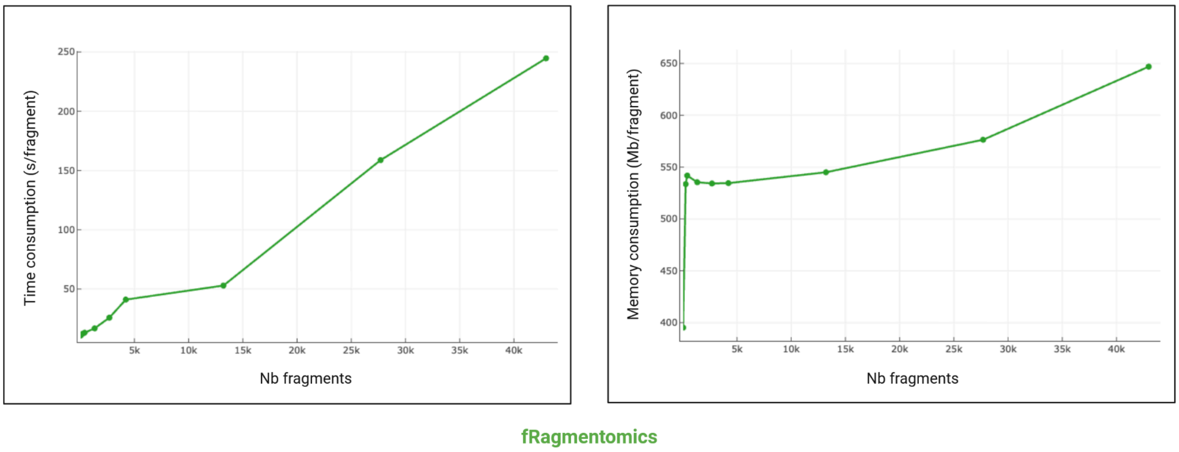

Supplementary Methods

S1. Mutation input normalization

fRagmentomics accepts several mutation input formats:

- A VCF file (standard format).
- A tab-delimited file with columns `chr`, `pos`, `ref`, `alt`
- A list of strings formatted as `chr:pos:ref:alt`

The files may be compressed or not. Internally, all representations are harmonized into a unified data frame using the following rules:

1. **Coordinate conventions:** both one-based and zero-based positions are supported. When the format is ambiguous, one-based indexing is assumed by default, consistent with VCF conventions.

2. **Normalization:** ambiguous or incomplete allele representations are standardized by left-aligning and trimming according to the rules defined in (Tan et al. 2015).

3. **Ambiguity resolution:** when available, the program can invoke `bcftools norm -m +both -d exact --check REF,ALT --fasta-ref <fasta>` to ensure consistency between the input alleles and the reference FASTA sequence.

Each normalized mutation is stored as a 4-column record: `chr`, `pos,` `ref`, `alt`.

Here are examples of formats accepted for input mutations.

**Simple Format**			
**Mutation**	**REF Column**	**ALT Column**	**POS Column**
Deletion of **"AT"**	AT	"", -, ., _, NA	Position of the first deleted base (A)
Insertion of **"CT"**	"", -, ., _, NA	CT	Position of the base *before* the insertion
**VCF-Style Padded Format**			
**Mutation**	**REF Column**	**ALT Column**	**POS Column**
Deletion of **"AT"** from "G**AT**"	GAT	G	Position of the anchor base (G)
Insertion of **"CT"** after "A"	A	ACT	Position of the anchor base (A)
SNV “A” to “G”	A	G	Position of the first base (A)

MNV “AT” to “GG”	AT	GG	Position of the first base (A)

S2. Read selection and windowing

After normalization, *fRagmentomics* subsets the BAM file using `Rsamtools::scanBam()` to extract reads overlapping a user-configurable genomic window around the mutation (controlled by the parameters `neg_offset_mate_search` and `pos_offset_mate_search)` and filter reads according to the `flag_bam_list` parameter.

The flag_bam_list parameter controls which reads are extracted from the BAM file by `Rsamtools::scanBam()`. By default, the following reads are excluded:

- Unpaired reads (`FLAG 0x1` required)
- Unmapped reads (`FLAG 0x4` and `FLAG 0x8` excluded)
- Secondary and Supplementary alignments (`FLAG 0x100` and `FLAG 0x800` excluded)

Reads not selected by `scanBam()` cannot be recovered in the output.

Selected reads are then subjected to additional quality checks. Fragments failing any of these checks are by default excluded from the output. However they can be recovered by setting `retain_fail_qc = TRUE`. First, read pairs with identical strand orientations are removed. Second, reads for which no mate is found in the search window are examined. This may occur for four reasons: (i) the read has no mate (does not happen if `FLAG 0x1` is requested); (ii) the mate is unmapped (does not happen if `FLAG 0x4` and `FLAG 0x8` are requested); (iii) the mate maps to a different chromosome; and (iv) the mate maps on the same chromosome but outside the queried window. A specific diagnostic message is reported in the `Fragment_QC` column for each case.

The size of the genomic window is controlled by the `neg_offset_mate_search` and `pos_offset_mate_search` parameters (default: ±600 bp). This window serves to retrieve reads overlapping the mutation as well as their mates, which may align nearby without covering the variant position. The default parameters are appropriate for human cfDNA data, for which the vast majority of fragments have sizes that do not exceed two or three times the modal fragment length of 167 bp representing a single nucleosomal complex (Snyder et al. 2016) Users may increase these values to recover longer fragments. Fragments are indexed by a unique `Fragment_ID = <QNAME>`.

S3. Fragment size computation

Conventional fragment length (`TLEN` field in BAM) is computed as:

TLEN = (Last aligned position of read 3’) - (First aligned position of read 5’) + 1

which ignores insertions, deletions, and soft-clipped bases. In *fRagmentomics*, fragment size is instead defined as:

Fragment Size = (Read 5' Length) + (Inner Distance) + (Shared Deletions in Overlap) - (Shared Insertions in Overlap) + (Read 3' Length)

With

1. **Inner Distance** = (Read 3' Inner Boundary) - (Read 5' Inner Boundary) - 1
2. **Read 3' Inner Boundary** = (Read 3' first aligned position) - (3' Left Soft-clips)
3. **Read 5' Inner Boundary** = (Read 5' first aligned position) + (5' Matched Bases) - 1 + (5' Deletions) + (5' Right Soft-clips)

The read lengths are set to exactly the number of sequenced bases, including soft-clipped bases unless the user activated the `remove_softclip` option. Of note, the inner distance calculation implemented in *fRagmentomics* allows for fragment sizes smaller than the individual read lengths as fragments shorter than the experimentally defined read length contain substantial inner soft-clipped bases making the inner distance a high negative number. Examples are illustrated in **Supplementary Figure S1**.

S4. Read genotyping

S4.1 Window definition

The comparison window defines the portion of the read that is compared to the corresponding region of the reference and mutated sequences. It represents the minimal segment necessary to unambiguously determine whether the read supports the wild-type (WT), mutant (MUT), or another (OTH) allele. Using strictly the minimum required length prevents adjacent variants from interfering with the mutation status assignment.

For SNVs and MNVs, the window includes the substituted base(s). An additional window extended by one base each side is used to detect potential larger events and sets a warning flag if necessary.

For indels, additional complexity arises from repetitive contexts where the inserted or deleted motif may occur multiple times within the reference. In this case, the algorithm expands the window dynamically beyond the mutation itself to include:

1. One **anchor base** immediately upstream of the mutation (`n_match_base_before = 1`). Let n_{before} be this number.

2. The **mutated motif** (inserted or deleted sequence). Let L_{motif} be the motif length.

3. One **downstream base** (`n_match_base_after = 1`). Let n_{after} be the number of downstream bases.

4. When the indel motif (e.g., the deleted or inserted sequence) occurs repeatedly in the reference genome, additional bases are included to ensure the window extends beyond the repeated context. The number of repeats is counted using the

`repeat_count` variable (let R be this number), which represents the total number of consecutive occurrences of the motif in the reference sequence, including the instance involved in the mutation itself. If the repeated motif is followed by a partial repetition (i.e., a prefix shared with the motif but not forming a full repeat), this overlap is quantified by `n_bases_shared_with_motif` (let n_{shared} be this number)

For insertions, comparison windows are:

- $\textbf{compare\_len}_{\textbf{WT}} = n_{before} + R \times L_{motif} + n_{shared} + n_{after}$
- $\textbf{compare\_len}_{\textbf{MUT}} = n_{before} + (R+1) \times L_{motif} + n_{shared} + n_{after}$

For deletions, comparison window is:

- $\textbf{compare\_len}_{\textbf{WT}} = \textbf{compare\_len}_{\textbf{MUT}} = n_{before} + (R-1) \times L_{motif} + n_{shared} + n_{after}$

Once the window is defined, the corresponding portion of the read covering this region is extracted and used for comparison. Examples are illustrated in **Supplementary Figure S4**.

S4.2 Sequence comparison

For each read overlapping a mutation, the observed read sequence over the window is compared to the reference sequence queried from the FASTA and the mutated sequence (constructed from applying `ref` → `alt` substitution or indel to the reference sequence).

Let S_r be the read sequence, S_{ref} the reference sequence, and S_{alt} be the mutated sequence over the window length. Then the result of the sequence comparison is one of the four following possibilities:

- **Wild-type (WT)** if $S_r = S_{ref}$ and $S_r \neq S_{alt}$
- **Mutated (MUT)** if $S_r \neq S_{ref}$ and $S_r = S_{alt}$
- **Other mutation (OTH)** if $S_r \neq S_{ref}$ and $S_r \neq S_{alt}$
- **Ambiguous (AMB)** if $S_r = S_{ref}$ and $S_r = S_{alt}$

S4.3 SNVs/MNVs genotyping

For SNVs and MNVs, since the information is not encoded in the CIGAR, read genotyping relies only on the sequence comparison (unlike indels, see Section S4.4 Indel genotyping). If the variant is not fully covered by the read, the comparison is considered incomplete and the read is labeled “ambiguous”. The bases surrounding the SNV/MNV position(s) are also examined to identify potential additional mutations. When such events are detected, an additional label “potentially OTH” is added to indicate that another alteration may be present.

Examples:

<u>SNV</u>
Variant: **Pos 5, A > G**

Reference: A G T A A G G G T C C
Mutant: A G T A **G** G G G T C C

Read	Read Mutational Status
A G T A A G G	“WT”
A G T A G G G	“MUT”
A G T A G T G	“MUT but potentially OTH”

MNV
Variant: **Pos 5, AT > GG**
Reference: A G T A A T G G T C C
Mutant: A G T A **G G** G G G T C C

Read	Read Mutational Status
A G T A A	“WT”
A G T A G	"AMB"
A G T A G G G	“MUT”
A G T A G G C	“MUT but potentially OTH”
A G T A A T G	“WT”

S4.4 Indels genotyping

For indels, we first verify whether the read is sufficiently long to cover the defined window. If it is not, the comparison is *incomplete*; otherwise, it is considered *complete*. We then inspect the CIGAR to determine whether the indel of interest or a different indel is present at the position of interest. A sequence comparison between the read and both the reference and mutant sequences is also performed.

If the indel of interest is detected from the alignment, the read is labeled “mutated”. If another indel is present, it is labeled “other”. When the CIGAR does not report any indel, the sequence comparison result is used directly, except when it indicates a mutation; in this case, we assign a “WT but potentially MUT” status. This distinction helps resolve discrepancies between paired reads from the same fragment while limiting false positives by avoiding sole reliance on sequence comparison when the alignment fails to report the indel in either read.

The sequence comparison result is also used to flag potential alignment errors in cases where an indel is detected in the CIGAR. The full set of rules for indel status assignment is provided below and illustrated with examples.

Read not covering the position of interest : Read Status = "NA"

Read covering the position of interest:

**Complete comparison** (reads long enough to distinguish MUT from WT without ambiguity)	
**Mutation found by CIGAR**	**Read Status**
MUT by comparison	"MUT"
WT by comparison	"MUT by CIGAR but potentially WT"
AMB by comparison	"IMPOSSIBLE"
OTH by comparison	"MUT by CIGAR but potentially OTH"
**Other found by CIGAR**	**Read Status**
MUT by comparison	"OTH by CIGAR but potentially MUT"
WT by comparison	"OTH by CIGAR but potentially WT"
AMB by comparison	"IMPOSSIBLE"
OTH by comparison	"OTH"
**WT found by CIGAR**	**Read Status**
MUT by comparison	"WT by CIGAR but potentially MUT"
WT by comparison	"WT"
AMB by comparison	"IMPOSSIBLE"
OTH by comparison	"OTH"
**Incomplete comparison** (reads not long enough to distinguish MUT from WT without ambiguity)	
**Mutation found by CIGAR**	**Read Status**
MUT by comparison	“MUT by CIGAR but AMB”
WT by comparison	"MUT by CIGAR but potentially WT"
AMB by comparison	"MUT by CIGAR but AMB"
OTH by comparison	"MUT by CIGAR but potentially OTH"
**Other found by CIGAR**	**Read Status**
MUT by comparison	"OTH by CIGAR but potentially MUT"

WT by comparison	"OTH by CIGAR but potentially WT"
AMB by comparison	"OTH by CIGAR but AMB"
OTH by comparison	"OTH"
**WT found by CIGAR**	**Read Status**
MUT by comparison	"WT by CIGAR but potentially MUT"
WT by comparison	"WT"
AMB by comparison	"AMB"
OTH by comparison	"OTH"

Examples:

Insertion
Variant: **Pos 4, A > AGG** (an insertion of 'GG' after anchor base 'A' at position 4)
Reference: A G T A G G G T C C
Mutant: A G T A **G G** G G G T C C

Insertion detected by CIGAR

Read	CIGAR	Read Mutational Status
A G T A G G G	6M2I1M	"MUT by CIGAR but AMB"
A G T A G G G G	6M2I2M	“MUT by CIGAR but AMB"
A G T A G G G G G	6M2I3M	"MUT by CIGAR but AMB"
A G T A G G G G G T	6M2I4M	“MUT”
A G T A G G G G G T	6M3I3M	"OTH by CIGAR but potentially MUT"

Insertion not detected by CIGAR

Read	CIGAR	Read Mutational Status
A G T A G G G	6M3M	"AMB"
A G T A G G G G	6M4M	"WT by CIGAR but potentially MUT"
A G T A G G G G G	6M5M	"WT by CIGAR but potentially MUT"
A G T A G G G G G G	6M6M	“OTH”

A G T A G G G G G T	6M6S	“WT by CIGAR but potentially MUT"

<u>**Deletion**</u>
Variant: **Pos 3, GACA > G** (a deletion of 'ACA' after anchor base 'G' at position 3)
Reference: G T G <u>**A C A**</u> A C A A G T C
Mutant: G T G A C A A G T C

Deletion detected by CIGAR

Read	CIGAR	Read Mutational Status
G T G A C A	3M3D3M	"MUT by CIGAR but AMB"
G T G A C A A	3M3D4M	"MUT by CIGAR but AMB"
G T G A C A A G	3M3D5M	"MUT"
G T G A C A A C	3M3D5M	"MUT by CIGAR but potentially WT"
G T G A C A A T	3M3D5M	"MUT by CIGAR but potentially OTH”

Deletion not detected by CIGAR

Read	CIGAR	Read Mutational Status
G T G A C A	6M	"AMB"
G T G A C A A	7M	"AMB"
G T G A C A A G	8M	"WT by CIGAR but potentially MUT"
G T G A C A A C	8M	"WT"
G T G A C A A G	5M3S	"WT by CIGAR but potentially MUT”

S5. Fragment genotyping

Fragments are assigned one of four statuses, WT, MUT, OTH, or N/I (non-informative), based on the two reads' statuses. The assignment rules are described in the table below where X and Y denote one of WT, MUT, or OTH (AMB is handled separately). The table does not list symmetric cases between read 1 and read 2 for conciseness.

Read 1	Read 2	Fragment	Description
X	X	X	Reads agree

X	Y	Non-informative (N/I)	Reads disagree
X	NA	X	Only one read covers the mutation.
X but potentially Y	X	X	Reads agree
X but potentially Y	Y	Y	Rescue agreement
X	AMB	X	Prioritize the non-ambiguous status
AMB	AMB	Non-informative (N/I)	No genotype is clearly supported
AMB	NA	Non-informative (N/I)	No genotype is clearly supported

Below are examples of fragment mutation statuses.

**Read 1**	**Read 2**	**Fragment_Status_Detail**	**Fragment_Status_Simple**
NA	MUT by CIGAR but AMB	MUT by CIGAR but AMB	MUT
AMB	MUT by CIGAR but AMB	AMB & MUT by CIGAR but AMB	MUT
OTH	OTH by CIGAR but potentially MUT	OTH & OTH by CIGAR but potentially MUT	OTH
WT	WT	WT	WT
WT	MUT by CIGAR but potentially WT	MUT by CIGAR but potentially WT & WT	WT
AMB	AMB	AMB	N/I
WT	MUT by CIGAR but potentially OTH	MUT by CIGAR but potentially OTH & WT	N/I

S6. Commands to reproduce outputs and plots in Fig.1

```
mut_file =
system.file("extdata/mutation","cfdna-egfr-del_chr7_55241864_55243
064_10k.mutations.tsv", package = "fRagmentomics")
bam_file =
system.file("extdata/bam","cfdna-egfr-del_chr7_55241864_55243064_10k.
bam", package = "fRagmentomics")
fasta_file =
system.file("extdata/fasta","hg19_chr7_55231864_55253064.fa",
package = "fRagmentomics")

run_fRagmentomics(
      mut = mut_file,
```

```
        bam = bam_file,
        fasta = fasta_file,
        apply_bcftools_ norm = TRUE
)

plot_size_distribution(
        df_results,
        vals_z = c("MUT", "WT"),
        show_histogram = TRUE,
        show_density = TRUE,
        x_limits = c(100,420),
        histo_args = list(alpha = 0.25),
        density_args = list(linewidth = 2),
        histogram_binwidth = 10,
        colors_z = c("#F6BD60", "#84A59D")
)

plot_freq_barplot(
        df_results,
        motif_size = 5,
        motif_type = "Both",
        col_z  = "Fragment_Status_Simple",
        vals_z = c("MUT", "WT"),
        colors_z = c("#F6BD60", "#84A59D")
)

plot_motif_barplot(
        df_results,
        motif_type = "Both",
        motif_start = "A",
        representation = "split_by_motif",
        vals_z = c("MUT", "WT"),
        colors_z = c("#F6BD60", "#84A59D")
)

plot_ggseqlogo_meme(
        df_results,
        motif_size = 3,
        motif_type = "Both",
        col_z  = "Fragment_Status_Simple",
        vals_z = c("MUT"),
        colors_z = c("#F6BD60", "#84A59D", "#FD96A9", "#083D77")
)
```

Supplementary References

Snyder, Matthew W., Martin Kircher, Andrew J. Hill, Riza M. Daza, and Jay Shendure. 2016. "Cell-Free DNA Comprises an In Vivo Nucleosome Footprint That Informs Its Tissues-Of-Origin." *Cell* 164 (1-2): 57–68.

Tan, Adrian, Gonçalo R. Abecasis, and Hyun Min Kang. 2015. "Unified Representation of Genetic Variants." *Bioinformatics (Oxford, England)* 31 (13): 2202–2204.
